# Subclinical hypothyroidism among infertile women at a tertiary hospital in South-West Nigeria

**DOI:** 10.4314/ahs.v22i2.51

**Published:** 2022-06

**Authors:** Olusoji E Jagun, Babatunde A Andu, Olatunbosun O Olawale

**Affiliations:** 1 Olabisi Onabanjo University Teaching Hospital, Sagamu

**Keywords:** Hypothyroidism, Infertility, Subclinical, TSH, Women

## Abstract

**Background:**

Overt thyroid dysfunction is an accepted cause of female infertility. Its milder form, subclinical hypothyroidism have also been implicated as a contributing factor to disturbed reproductive function.

**Objective:**

To determine the contribution of subclinical hypothyroidism (SCH) to the burden of infertility.

**Methodology:**

This is a cross sectional, comparative study of One hundred and twenty women with primary or secondaryinfertility who presented for evaluation at gynaecological clinic and controls which are clients that came to access Family planning services.

**Results:**

The prevalence of infertility among gynaecological patients seen in the clinic was 38.8% (192/495). The prevalence of SCH among the studied infertile women was 11.7% (7/60) compared with 3.3% (2/60) of the control group (p=0.222). The commonest type of infertility was secondary, 76.7% (46/60). All thestudied infertile women with SCH presented with secondary infertility. There was an observed statistically significant difference in the mean serum TSH (3.19±4.38mIU /L vs 1.60±1.22mIU /L) and FT3, FT4 (0.29±0.074ng/dl vs 0.95±0.16ng/dl and 0.33±0.071ng/dl vs 1.09±0.19ng/dl respectively)

**Conclusion:**

Subclinical hypothyroidism was found to be higher among infertile women but this finding was not statistically significant, therefore, the routine screening for SCH among infertile women might be unnecessary

## Introduction

In Africa, children are the fabric of any society, without which no significant social and economic progress is considered worthwhile.^1^ The prevalence of infertility is estimated to range between 10% and 15% and has remained stable over the decades in the Western world.^2^,^3^ In Africa, the prevalence of infertility has been rising significantly. The reported infertility rates among married couples in African countries vary considerably, ranging from 15% to 30%.^4^

Hypothyroidism is a very common but subtle endocrinological problem with a variety of changes in reproductive functions including delayed onset of puberty, menstrual disorders, anovulatory cycles, infertility and reproductive wastage when pregnancy is achieved.^5^,^6^ Its milder form, subclinical hypothyroidism (SCH), characterized by mildly elevated thyroid stimulating hormone (TSH) levels (4.5–20mIU/L), normal free triiodothyronine (0.2–0.5ng/dl) and normal free thyroxine levels (0.76–2.24ng/dl), has also been implicated as a contributing factor to disturbed reproductive function^5^. The menstrual pattern is influenced by thyroid hormones directly through impact on the ovaries and indirectly through impact on sex hormone binding globulin (SHBG), prolactin and gonadotrophin releasing hormone (GnRH) secretion and coagulation factors.^2^ Previous studies have suggested that subclinical hypothyroidism is one of the important thyroid dysfunctions resulting in infertility and may be associated with ovulatory dysfunction.^6^,^7^,^8^ The reported prevalence of subclinical hypothyroidism among unselected infertile women was 14% 9 against the 2–4% reported among the general population.^10^

Subclinical hypothyroidism has long been considered as an infertility factor.^11^ In a particular study, it was observed that the mean duration of infertility was significantly longer in subjects with subclinical hypothyroidism when compared with those who had normal levels of thyroid hormones.^2^ Similarly, another study observed that women with elevated serum TSH levels had lower pregnancy rate than those with normal circulating levels.^11^ These findings therefore suggest that subclinical hypothyroidism may have impact on female fertility. Hence this comparative cross sectional study is designed to determine the association of subclinical hypothyroidism with female infertility in our environment. Findings from this study may therefore determine the necessity for routine screening for subclinical hypothyroidism among women presenting with fertility.

## Methodology

### Study setting

The study was conducted in an urban area at the gynaecologicaland family planning clinics of the Department of Obstetrics and Gynaecology of Olabisi Onabanjo University Teaching Hospital (OOUTH), Sagamu, Ogun state, South western, Nigeria. This is a public facility that serves as referral center to many hospitals in the state and neighbouring states.

Gynaecological services are provided for our women by nurses, resident doctors undergoing specialist gynaecological training and consultant gynaecologist. The assays of the serum TSH, free triiodothyronine and free thyroxine level were done in conjunction with the Chemical pathologist at the laboratory service department of the hospital.

### Study population

The study population consists of women of reproductive age (15–49 years) who had been married for at least one year with no obvious history of thyroid disease presenting with ovulatory factor infertility and parous (fertile) women at Family Planning clinic without history of thyroid disease as controls at OOUTH, Sagamu.

### Study Design

This was a comparative (cross sectional) study of consecutive samples of women of reproductive age withovulatory factor infertility as cases while the control group were parous (fertile) women attending family planning clinic. The controls were matched with age of the infertile population. Women with Overt hypothyroidism, overt hyperthyroidism, history of previous thyroid surgery, history of previous or recent treatment with thyroid medication and women with any obvious cause of infertility other than anovulation were excluded from the study.

Overt hypothyroidism is seen as an increase in serum TSH levels (> 20 mIU/L) and decrease in serum free T4 concentrations while subclinical hypothyroidism is characterized by normal serum free thyroxine concentration with elevated serum TSH concentrations ranging between 4.5 mIU/L and 20 mIU/L.^12^ The reference value for serum TSH concentration in normal individual ranges between 0.45 and 4.5mIU/L.^12^

Data collected include socio-demographic data, duration of marriage and infertility, history of any previous pregnancies and outcome, menstrual history and any possible abnormalities associated with it. In addition, serum was collected for the thyroid hormones assays and all these were recorded on the data collection form.

### Data analysis

Data were analyzed using Statistical Package for Social Science (SPSS) windows version 23. Demographic and baseline variables were summarized using descriptive statistics expressed as mean (standard deviation) or median (range) for continuous variables and percentages for categorical variables. Associations between variables were determined using independent t-test, Chi square and ANOVA as appropriate. The level of statistically significance was set at p-value <0.05.

### Ethical consideration

Ethical approvalfor the study was obtained from the Health Research Ethics Committee of Olabisi Onabanjo University Teaching Hospital, Sagamu (OOUTH/REC/038/2015). Consent was taken from the women and they were assured that non consent will not affect the level of management in any way

## Result

One hundred and ninety-two infertile (ovulatory and other causes) women were seen among the 495 gynaecological cases that presented within the six months period of study giving a prevalence of 38.8%. Table 1 shows the socio-demographic characteristics of the infertile women and the control group. All the studied participants were within the reproductive age limit of 18–45 years, with the majority in the age range of 31–40 years; 53.3% for cases and 58.3% for controls. The mean ages of cases and controls were 34.23±6.80 years and 34.72±6.12 years respectively.

**Table 1:** Comparison of socio-demographic features of cases and controls

Variables	Infertile women n=60(%)	Control n=60(%)	X^2^ Value	p-Value
**Age** (years)				
</=20	2(3.3)	0(0.0)		
21–30	16(26.7)	12(20.0)	3.873	0.276
31–40	32(53.3)	35(58.3)		
41–50	10(16.7)	13(21.7)		

**Educational status**				
None	0(0.0)	6(10.0)		
Primary	9(15.0)	6(10.0)	9.827	0.020
Secondary	28(46.7)	22(36.7)		
Tertiary	23(38.3)	26(43.3)		

**Religion**				
Christianity	40(66.7)	45(75.0)	1.008	0.315
Islam	20(33.3)	15(25.0)		

**Tribe**				
Yoruba	48(80.0)	52(86.7)		
Igbo	6(10.0)	5(8.3)	1.579	0.664
Hausa	5(8.3)	2(3.3)		
Others	1(1.7)	1(1.7)		

**Occupation**				
Unskilled	8(13.3)	8(13.3)		
Semi-skilled	30(50.0)	26(43.3)	0.847	0.838
Skilled	15(25.0)	16(26.7)		
Professional	7(11.7)	10(16.7)		

**Body Mass Index**				
Underweight	8(13.3)	2(3.3)		
Normal	21(35.0)	37(61.7)	10.996	0.012
Overweight	17(28.3)	14(23.3)		
Obese	14(23.3)	7(11.7)		

All the women in both groups had some form of education with most of them having secondary and tertiary education (85% for cases and 80% for controls). Most of the participants were semi-skilled workers (50% for cases and 43.3% for controls) and there was no statistically significant difference in the job they engage in (P=0.838). There was an observed statistically significant difference in the Body mass index of both cases and controls (P=0.012), 28.3% versus 23.3% were overweight among cases and controls and 23.3% versus 11.7% were obese among cases and controls.

Table 2, shows the mean age and mean duration of infertility among cases. Women with primary infertility had mean age of 30.00±6.61 years, mean serum TSH, free T3 and free T4 of 2.21±1.20 mIU/L, 0.27±0.05ng/dl and 0.90±0.15ng/dl respectively and mean duration of infertility of 2.79±1.89 years. Women with secondary infertility had mean age of 35.52±6.38 years, mean serum TSH, free T3 and free T4 of 3.48±4.93 mIU/L, 0.29±0.08ng/dl and 0.97±0.16 ng/dl respectively and mean duration of infertility of 4.57±3.87 years. There was an observed statistically significant difference between the mean ages among the women with primary and secondary infertility (P= 0.007).

**Table 2:** The mean age and mean duration of infertility among cases

Variables	Primary Infertility (n=14) Mean±SD	Secondary Infertility (n=46) Mean±SD	t-Test	p-Value
Mean age (years)	30.00±6.61	35.52±6.38	-2.810	0.007
Mean duration of infertility (years)	2.79±1.89	4.57±3.87	-1.653	0.104
Serum TSH (mIU/L)	2.21±1.20	3.48±4.93	-0.955	0.344
Serum fT3 (ng/dl)	0.27±0.05	0.29±0.08	-0.618	0.539
Serum fT4 (ng/dl)	0.90±0.15	0.97±0.16	-1.583	0.119

The mean serum TSH, free T3 and free T4 among cases were 3.19±4.38 mIU/L, 0.29±0.074ng/dl and 0.95±0.16ng/dl respectively while among controls were 1.60±1.22 mIU/L, 0.33±0.071ng/dl and 1.09±0.19ng/dl respectively (Table 3). Thus, these variables were statistically significantly different among the cases and controls (P=0.008 & 0.001).

**Table 3:** Mean ± SD of serum group, and association of subclinical hypothyroidism with cases and controls

Variable	Infertile women(n=60)	Control(n=60)	t-Test /χ^2^	p-value
Serum TSH±SD mIU/L	3.19±4.38	1.60±1.22	2.709	0.008
Serum fT3±SD ng/dl	0.29±0.074	0.33±0.071	-3.570	0.001
Serum fT4±SD ng/dl	0.95±0.16	1.09±0.19	-4.307	0.001

Subclinical Hypothyroidism	Freq.(%)			
Euthyroid	52(86.7)	57(95.0)		
Sub-Hypo	7(11.7)	2(3.3)	3.007	0.222
Sub-Hyper	1(1.7)	1(1.7)		

Sub-Hypo: Subclinical hypothyroidism Sub-Hyper: Sub-clinical hyperthyroidism

**Figure 1:**
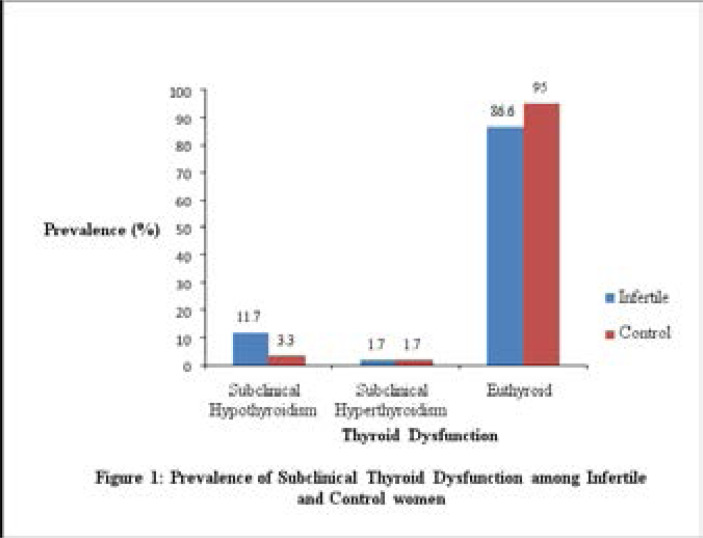
Prevalence of Subclinical Thyroid Dysfunction among Infertile and Control women

Out of the 60 cases recruited, 7(11.7%) were subclinical hypothyroid, 1(1.7%) was subclinical hyperthyroid and 52(86.6%) were euthyroid. (Figure 1). Out of the 60 controls recruited, 2(3.3%) were subclinical hypothyroid, 1(1.7%) was subclinical hyperthyroid and 57(95%) were euthyroid. (Fig 1). In women with infertility, the prevalence of subclinical hypothyroidism was 11.7 % (7/60) while a prevalence of 3.3% (2/60) was observed in the control group. (Table 3).

Seven (15.2%) women with subclinical hypothyroidism were seen to present with secondary infertility while none presented with primary infertility. This observed difference was not statistically significant (P=0.100).

## Discussion

Infertility is a common health problem with devastating psychosocial consequences on the affected couples especially in Africa.^13^ In this study, the prevalence of infertility among gynaecological patients that presented at the clinic was 38.8% (192/495), much more than the 14.8% earlier observed at the same facility fourteen years ago.^14^ This observed increase can be attributed to high prevalence of sexually transmitted infections and inadequate treatment of such infections, complication of unsafe abortion and puerperal sepsis.^14^,^5^ This observed high prevalence is comparable with 44.2% reported at Ikire, 47.7% at Osogbo and lower than 59.4% at Ile Ife all in South western Nigeria.^1^

The commonest type of infertility was secondary, seen in 76.7% (46/60) of participants while primary infertility was observed in 23.3% (14/60). The predominance of secondary infertility in this study is consistent with several studies in sub-Saharan Africa.^14^,^18^ This is however different from the trend in the developed countries where primary infertility is higher. The pattern in the developing countries is probably due to higher chance of acquired diseases of the reproductive tract from sexually transmitted infections, unsafe abortions and puerperal sepsis.^14^ The mean duration of infertility in this study was 3.15±2.28 years comparable to 3.38 years earlier reported at the same centre by Olatunji and Sule Odu,^14^ which is also similar with the mean duration of less than 5 years reported in other studies.^6^,^16^ The shorter duration of infertility observed in this study might be due to the fact that most of the infertile women in this study are literate, probably earn well and seek fertility treatment early.

Hypothyroidism is the most common endocrinological problem affecting women who present with ovulatory dysfunction resulting in infertility.^5^ Its milder form, subclinical hypothyroidism characterized by mildly elevated thyroid stimulating hormone (TSH) levels, normal free T4 and free T3 may also contribute to disturbed reproductive function.^5^

The mean serum TSH, free T3 and free T4 levels were higher among the infertile women than the values in the fertile women. This trend was statistically significant (P= 0.008 for serum TSH) and (P= 0.001for serum T3 and T4). A similar trend was reported by Biradar et al,^6^ where serum TSH, T3 and T4 were found to be significantly increased in infertile females compared to control group. Majority of the patients among the infertile women had normal thyroid function, i.e. euthyroid 86.7% (52/60) which shows that other causes of infertility could be responsible for this female infertility. This result is comparable with studies by Rijal et al.^7^ and Pushpagiri et al.^17^

The impact of hypothyroidism on ovulation and menstrual function is related to numerous interactions of the thyroid hormones with the female reproductive system, thus finally leading to infertility.^6^,^18^ In hypothyroidism, increased TRH production leads to hyperprolactinaemia and altered GnRH pulsatile secretion. This leads to a delay in LH response and inadequate corpus luteum leading to abnormal follicular development and anovulation.^6^,^18^ In this study, it was observed that 11.7% of the studied infertile women were suffering from subclinical hypothyroidism compared to the control group where a prevalence of 3.3% was found. The prevalence of subclinical hypothyroidism of 11.7% observed among the infertile women in this study is similar to 12% reported by Biradar et al^6^;but lower than 53% reported by Sridevi et al;^19^ 43.3% reported by Habbu et al,^20^ and 23.4% reported by Emokpae et al.^13^ It is of note thatEmokpae studied infertile women with hyperprolactinemia which could have accounted for the observed difference, as prolactin has been shown to be positively correlated to TSH.However, a lower prevalence of 4.6% was reported by Grassi et al.^6^ The higher prevalence found in some of these studies might be due to the fact thatthe large population of infertile women studied had ovulatory dysfunction. The findings from this study is also consistent with areas of high iodine deficiency in India and much higher than the global average.^10^ The fertile control group has a prevalence of (3.3%) which is comparable with global average, this therefore means that further studies will be necessary to determine the iodine levels in the general population. The observed differences in these studies were not statistically significant. This observation might be due to small population of studied participants.

Seven (15.2%) women with subclinical hypothyroidism presented with secondary infertility while none presented with primary infertility. The data shows that subclinical hypothyroidism was relatively more common in women with secondary infertility. This finding is not consistent with the study by Pushpagiri et al.^17^ where the subclinical hypothyroidism was more common in primary infertile women because endocrinological/ovulatory dysfunctions were responsible for most of the primary infertility cases seen.

Although the prevalence of subclinical hypothyroidism was found to be higher among infertile women compared to fertile women in OOUTH, Sagamu, this finding was not statistically significant (P=0.222). This might make routine assays of TSH in infertile women unnecessary.

The study is limited by the small sample size and it was not multicentered, secondly, it was a cross-sectional study and you cannot objectively assert that SCH onset had a causal relationship with infertility

## Conclusion

Subclinical hypothyroidism among infertile women evaluated was 11.7% compared to 3.3% in their fertile counterparts but this finding is not statistically significant therefore the routine screening for subclinical hypothyroidism among women being evaluated for infertility might be unnecessary. Further multicentre studies involving large numbers of infertile women is recommended in order to validate this finding.
